# Developing a quality measure to assess use of antibiotic medications for respiratory conditions

**DOI:** 10.1017/ash.2022.328

**Published:** 2023-01-17

**Authors:** Brandi L. Melville, Taylor Musser, Ezra Fishman, Danielle Rainis, Sepheen C. Byron

**Affiliations:** National Committee for Quality Assurance, Washington, DC

## Abstract

**Objective:**

Antibiotics are essential medications for treating life-threatening infections. However, incorrect prescribing can lead to adverse events and contribute to antibiotic resistance. We sought to develop a utilization quality measure that could be used by health insurance plans to track overall prescribing for respiratory conditions.

**Design:**

A consensus-based process that included evidence review, testing, and stakeholder input was used to develop a measure and assess its usefulness for the Healthcare Effectiveness Data and Information Set (HEDIS), a national quality measurement tool.

**Methods:**

Guidelines and literature were reviewed to establish the rationale for the measure. The measure was tested in claims data for commercial, Medicaid and Medicare Advantage enrollees to assess feasibility of collecting and reporting needed information. The measure was vetted with multistakeholder advisory panels and posted for public comment to solicit wide input on relevance and usability.

**Results:**

Respiratory conditions are frequent reasons for outpatient care in the data assessed. On average, across all lines of business, the measure revealed that approximately one-third of outpatient visits for respiratory conditions are followed by antibiotics. Stakeholders supported the measure as a tool for monitoring antibiotic prescribing across health plans alongside existing measures that assess inappropriate prescribing for specific conditions. The final measure assesses the number of antibiotic prescriptions dispensed across all outpatient respiratory-related encounters at a health-plan level.

**Conclusions:**

The measure on antibiotic prescribing for respiratory conditions was relevant, feasible, and useful. Stakeholders strongly supported the newly developed measure and recommended its integration into HEDIS.

Antibiotics are essential medications for individuals with life-threatening infections. However, any time they are prescribed, they can lead to adverse events and contribute to antibiotic resistance, including >2.8 million antibiotic-resistant infections occurring in the United States.^1^ The consequences of antibiotic resistance include limited treatment options for certain conditions, healthcare-system strain from longer hospitalizations, and an inability to produce new antibiotics at a pace to match emerging infections.^2^ Furthermore, the coronavirus disease 2019 (COVID-19) pandemic prompted increased use of telemedicine, which merits attention given physical examination limitations and other potential impediments to antibiotic stewardship strategies.^3,4^

Ambulatory settings account for a large proportion of antibiotic prescriptions, with marked variation across primary, retail, urgent-care and emergency-care providers.^5,6^ Thus, health insurance plans are uniquely positioned to establish antibiotic stewardship programs. Health plan administrators have access to data on their enrollees’ healthcare encounters, diagnoses, and treatments. The ability of health plan administrators to leverage those data across various settings is particularly beneficial for antibiotic stewardship efforts.

Measurement is 1 of the 4 Core Elements of Outpatient Antibiotic Stewardship.^7^ Quality measures facilitate prospective auditing of prescribing trends and can assess the impact of interventions such as provider education or prior-authorization processes.^8^ National quality measures related to judicious antibiotic use are available in the Healthcare Effectiveness Data and Information Set (HEDIS). HEDIS is used to evaluate the quality of care for >200 million individuals covered by commercial, Medicaid, and Medicare managed-care plans.^9^ HEDIS is used to assess whether health plan administrators ensure that their members receive recommended services, treatments, and care management.

In HEDIS, quality measures assess appropriate antibiotic prescribing for 3 targeted clinical diagnoses: bronchitis, upper respiratory infection, and pharyngitis. These conditions, along with all other respiratory conditions, account for >30% of inappropriate antibiotic prescribing.^10^ The performance of these measures has shown modest improvement between 2010 and 2019.^11–13^ However, more information may assist health plan administrators to discern whether improvement is due to better antibiotic treatment decisions or whether it is a reflection of factors unrelated to judicious prescribing, such as misdiagnosis or overdiagnosis.^14^ Although assessing antibiotic prescribing for targeted conditions is important, understanding prescribing within a health plan across a broader range of respiratory conditions can complement stewardship efforts.

Measuring antibiotic utilization for all respiratory conditions presents an opportunity to further analyze prescribing practices and the factors that may contribute to high antibiotic prescribing in outpatient settings. These trends may be influenced by patient desire for antibiotics, provider diagnosing, and coding practices.^15^ Pairing data on condition-specific antibiotic prescribing with data on antibiotic prescribing across all respiratory conditions may provide the additional context to advance antibiotic stewardship. Given the prevalence of antimicrobial resistance, high antibiotic prescribing rates, and an increased emphasis on antibiotic stewardship, we sought to develop a utilization measure that provides additional information to complement the existing portfolio of HEDIS antibiotic prescribing measures. Here, we describe the process undertaken to develop, test, and achieve stakeholder consensus for a measure assessing overall utilization of antibiotics for respiratory conditions.

## Methods

A development process that considers clinical evidence, data analysis and multistakeholder input was used.^16^ The process is designed to ensure that any resulting measure exhibits desirable attributes of relevance, scientific soundness, and feasibility. Relevance refers to the importance of the measure to its potential users. Scientific soundness refers to how closely the measure reflects evidence-based guidelines. Feasibility refers to whether the measure can be reported without undue burden. To ensure that a wide range of perspectives were considered, a multistakeholder panel was convened and served as the advisory group to the process.

### Conceptualization

The process begins with conceptualizing a measure using scientific evidence and input from the advisory panel. Representatives from health plans, clinicians, public health, and the scientific community were identified by the project team based on the individual’s expertise in the field and were recruited for the panel. In some cases, recruited individuals were participants of prior antibiotics-related measure development projects. As a condition of appointment, potential conflicts of interest were assessed for all panel members.

The panel provided feedback on key measurement characteristics, including how to define the population to be measured, the range of respiratory conditions to be assessed, and other characteristics that could affect the measure’s usefulness to clinicians, health plan administrators and policy makers. In addition, a separate technical panel composed of quality measurement experts was consulted. This group is a standing advisory panel maintained by the National Committee for Quality Assurance, which stewards HEDIS; the group comprises representatives from health plans, measure auditors, and federal and state measurement program leads. The panel provided input on the feasibility of a respiratory-condition–focused antibiotic prescribing measure based on data sources and analytic capabilities available to health plan administrators.

### Specification

Key features of the draft measure were developed and specified for testing. The lists of respiratory conditions, competing diagnoses, and comorbid conditions were primarily developed by reviewing and aligning them to the existing antibiotics-related measures in HEDIS. The lists were vetted by the advisory panels. A value set using standardized *International Classification of Disease, Tenth Revision* (ICD-10) and *Systematized Nomenclature of Medicine* (SNOMED) codes was produced.

- *Defining respiratory conditions.* Respiratory conditions were defined as viral or bacterial infections and other illnesses of the upper and lower respiratory tract. These conditions served to identify episodes eligible for the measure.
- *Defining competing diagnoses*. Competing conditions were defined as a non–respiratory-antibiotic–indicated diagnosis, such as urinary tract infection, present on the same day as the respiratory condition encounter or ≥3 days after. These are conditions outside respiratory conditions that may warrant treatment with antibiotic medications and therefore should result in the encounter being removed from the measure.
- *Defining comorbid conditions*. Comorbid conditions were defined as diagnoses in the 12 months prior to the respiratory-condition encounter for immunocompromising conditions (eg, human immunodeficiency virus, cancer, disorders of the immune system, or chronic conditions) that may be managed with unique clinical discretion (eg, emphysema or chronic obstructive pulmonary disease).

### Field testing

Once the measure was conceptualized, testing was conducted to provide empirical evidence of the measure’s feasibility and performance rates, as well as to inform other technical features of the measure. Specifically, analyses examined the following: (1) the volume of outpatient visits for respiratory conditions (ie, the denominator); (2) the frequency of potential conditions that would exclude an encounter from the denominator; (3) the volume of antibiotic prescriptions dispensed for respiratory conditions (ie, the numerator); and (4) performance rates according to proposed measure specifications.

### Data sources

Analyses for Medicare and commercial lines of business were conducted using deidentified administrative claims data from the Optum Labs Data Warehouse, which includes medical and pharmacy claims, laboratory results, and enrollment records for commercial and Medicare Advantage (MA) enrollees. The database contains longitudinal health information on enrollees and patients representing a mixture of ages, ethnicities, and geographic regions across the United States.^17^ The testing was conducted from June through October 2020 using data from the National View that represented healthcare utilization occurring in calendar years 2018 and 2019. The data included index outpatient visits (denominator events) occurring from January 1, 2019, through December 31, 2019, and antibiotic dispensing dates (numerator events) occurring from January 1, 2019, through January 3, 2020. All analyses for the Medicare and commercial populations were conducted using R software.

Medicaid testing was conducted using the IBM MarketScan Multistate Medicaid Database, which contains medical, surgical, and prescription drug claims for >47 million Medicaid enrollees from multiple states.^18^ A unique enrollee identifier was assigned to each individual consistent across years. To maintain patient confidentiality and to adhere to data use agreements, results were not available at the health-plan level. The testing was conducted from October through December 2020, using data on index outpatient visits (denominator events) occurring from January 1, 2018, through December 31, 2018, and antibiotic dispensing dates (numerator events) occurring from January 1, 2018, through January 3, 2019. All analyses for the Medicaid population were conducted using WPS Analytics (a SAS-like platform).

### Analysis

Descriptive analyses for all lines of business were performed at the population level to determine counts and proportions of outpatient visits based on member age and respiratory condition diagnosis. At the health-plan level, the distribution of outpatient visits for respiratory conditions was also calculated for the Medicare and commercial lines of business. Performance rates for the measure were calculated for each product line. The denominator assessed was the count of eligible outpatient visits for respiratory conditions; the numerator assessed was the number of visits for which an antibiotic dispensing event occurred within 3 days. Because data were based on completed claims, numerator events were measured as antibiotics dispensed. This method produced a rate expressed as the percentage of episodes for members ages 3 months and older with a diagnosis of a respiratory condition that resulted in an antibiotic dispensing event.

Visits were excluded if they resulted in an inpatient stay, if they were associated with a member who had a history of the defined comorbid conditions or competing diagnoses, or if they were dispensed an antibiotic in the 30 days prior to the qualifying event. The impact of excluding these visits was assessed by examining the reduction in frequency and proportion of total visits that occurred after implementing the exclusions. Visits qualified for inclusion if they were conducted among members who were continuously enrolled in a health plan from 30 days prior to the visit date through 3 days after the visit date, for a total of 34 days. For members who had >1 visit in a 31-day period, only the first eligible visit was counted. This method of deduplicating visits aligns with the 3 existing HEDIS measures assessing antibiotic use for bronchitis, upper respiratory infection, and pharyngitis.^19–21^ Also in line with the existing HEDIS measures, members who used hospice services were excluded.

### Public comment

The draft technical specifications, measure rationale and testing summary were posted for a 30-day public comment period between February and March 2021. Broad feedback was sought to understand the measure’s relevance, perceived feasibility, and usefulness to stakeholders. All comments were reviewed and discussed with the advisory panels.

## Results

### Field test results

*Event characteristics for respiratory conditions.* Results indicate that respiratory conditions are frequent reasons for outpatient care in the databases assessed (Table 1). Children and adolescents accounted for the largest proportion (71%) of respiratory-related visits in Medicaid. Among the commercially insured, rates were more evenly distributed among the age strata up to age 64 years. In Medicare, respiratory-condition–related visits were driven by beneficiaries ages 65–74 years. Among respiratory-related visits, the primary clinical drivers differed by population. Outpatient visits can include multiple respiratory condition diagnoses; therefore, individual visits may be counted in multiple condition categories.

**Table 1.** Characteristics of Outpatient Visits for Respiratory Conditions by Line of Business

Characteristic	Respiratory Condition Outpatient Visits, No. (%)					
	Medicare^a^		Commercial^a^		Medicaid^b^	
Total visits	4,827,022	(100.0)	3,022,301	(100.0)	4,600,566	(100.0)
**Age**						
3 mo–9 y	…	(0.0)	735,637	(24.3)	2,363,061	(51.4)
10–17 y	…	(0.0)	333,827	(11.0)	916,468	(19.9)
18–34 y	18,824	(0.4)	584,470	(19.3)	612,805	(13.3)
35–49 y	119,907	(2.5)	593,466	(19.6)	395,963	(8.6)
50–64 y	697,816	(14.5)	597,171	(19.8)	295,181	(6.4)
65–74 y	2,113,421	(43.8)	118,277	(3.9)	14,059	(0.3)
75–84 y	1,407,733	(29.2)	41,014	(1.4)	2,541	(0.1)
85+ y	469,320	(9.7)	18,439	(0.6)	488	(<0.1)
**Clinical condition^c^**						
Epiglottitis	208	(0.0)	127	(0.0)	79	(<0.1)
Mastoiditis	2,113	(0.0)	773	(0.0)	645	(<0.1)
Pneumonia	256,148	(5.3)	67,529	(2.2)	50,051	(1.1)
Streptococcal pharyngitis	11,341	(0.2)	118,649	(3.9)	292,892	(6.4)
Otitis media	74	(0.0)	46	(0.0)	549,051	(11.9)
Pharyngitis and tonsillitis	132,066	(2.7)	451,894	(15.0)	754,561	(16.4)
Sinusitis	459,260	(9.5)	466,756	(15.4)	379,964	(8.3)
Asthma	547,076	(11.3)	330,236	(10.9)	644,540	(14.0)
Bronchitis	446,542	(9.3)	227,829	(7.5)	297,067	(6.5)
Influenza	44,860	(0.9)	121,889	(4.0)	197,256	(4.3)
Laryngitis tracheitis	13,955	(0.3)	39,969	(1.3)	59,824	(1.3)
Rhinitis	374,985	(7.8)	278,364	(9.2)	368,901	(8.0)
Serous otitis media	34,081	(0.7)	79,292	(2.6)	138,500	(3.0)
Upper-respiratory infection	318,428	(6.6)	523,731	(17.3)	991,835	(21.6)
**Visit type**						
Outpatient	3,925,020	(81.3)	2,739,534	(90.6)	3,537,908	(76.9)
Telephone	90	(0.0)	6,195	(0.2)	…	(0.0)
Online assessment	42	(0.0)	296	(0.0)	11	(0.0)
Observation stay	87,608	(1.8)	12,848	(0.4)	13,549	(0.3)
Emergency department	814,262	(16.9)	263,428	(8.7)	1,049,098	(22.8)

a Medicare and commercial respiratory condition visits were evaluated without applying continuous enrollment and deduplication.

b Medicaid respiratory condition visits were evaluated after continuous enrollment and deduplication.

c Outpatient visits can include multiple respiratory condition diagnoses. Visits may be counted in multiple condition categories. Visits with diagnoses not mapped to the condition categories were omitted. Therefore, visits will not sum to the total.

*Distribution of respiratory conditions across Medicare and commercial health plans.* We evaluated the frequency of respiratory condition visits across individual health plans for the Medicare and commercial product lines; data for Medicaid did not permit plan-level calculations. Table 2 shows results of this analysis, in which we sought to understand whether focusing the measure on respiratory conditions and whether using an episode-based denominator yields small numbers, hindering reporting feasibility. The distribution of outpatient visits for respiratory conditions across 47 Medicare Advantage and 28 commercial health plans indicates that nearly all have at least 30 respiratory-condition–related visits during the year. Health plans with <30 visits were found to have small overall member enrollments.

**Table 2.** Distribution of Outpatient Visits for Respiratory Conditions Across Health Plans

Line of Business	No. of Plans	Percentile Count of Visits								
		Average	Standard Deviation	Minimum	10th	25th	50th	75th	90th	Maximum
Medicare	47	18,463.6	39,008.4	20.0	249.6	1,029.0	3,666.0	20,011.0	41,977.4	230,548.0
Commercial	28	45,402.6	148,363.6	26.0	117.6	298.0	2,950.0	8,973.0	56,793.6	760,130.0

Excluding conditions for which an antibiotic prescription may be warranted removed differing numbers of events depending on the line of business. Across the commercial population, the exclusions collectively omitted ∼43% of visits initially captured. Excluding visits with a competing diagnosis had the largest impact, removing ∼25% of visits. For the Medicare population, the exclusions omitted >75% of visits. Excluding visits among members with a history of select comorbidities was the primary driver, excluding ∼58% of visits. For Medicaid, the comorbid condition exclusion removed 9.1% of visits. Similar small impacts were seen among the prior antibiotic use and competing diagnosis exclusions.

#### Antibiotic dispensing

**Fig. 1.**
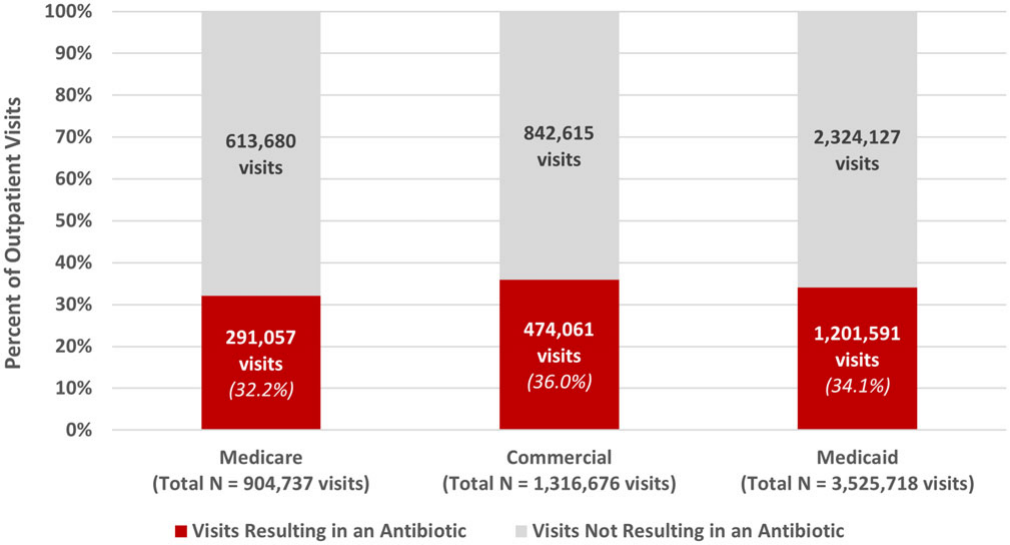
Antibiotic prescribing among outpatient visits for respiratory conditions by line of business.

Results at the national level following removal of exclusions are shown in Figure 1. All lines of business demonstrated similar national rates of antibiotic dispensing for respiratory condition visits.

The distribution of antibiotic dispensing rates across plans was similar for the Medicare and commercial product lines (Table 3). The median measure rate across Medicare plans was 31%, with an interquartile range of 7%. The median measure rate across commercial plans was 37%, with an interquartile range of 5%.

**Table 3.** Percent of Outpatient Respiratory Encounters that Resulted in an Antibiotic Dispensing Event Across Medicare and Commercial Health Plans

Product Line	No. of Plans	Average	Standard Deviation	Distribution, %						
				Minimum	10th	25th	50th	75th	90th	Maximum
Medicare	47	30.4	7.2	8.6	22.7	27.2	30.6	34.6	39.3	43.3
Commercial	28	35.2	8.0	0.0	30.4	33.2	36.7	38.1	40.8	46.2

Table 4 shows rates of antibiotic dispensing for respiratory condition outpatient visits in the Medicaid population. Rates of antibiotic dispensing were ∼34% for children and adults aged ≤65 years. The rate was 24% for adults aged ≥65 years. Rates were similar for male and female members.

**Table 4.** Number of Respiratory Encounters that Resulted in an Antibiotic Dispensing Event Across the Medicaid Population

Member Characteristic	Respiratory Conditions, No. of Events	Antibiotics Dispensed, No. of Events	Rate %
**Age**			
3 mo–17 y	2,688,017	893,148	33.2
18 – 64 y	832,402	307,180	36.9
≥65 y	5,299	1,263	23.8
**Sex**			
Male	1,570,302	510,664	32.5
Female	1,955,416	690,927	35.3

### Stakeholder feedback

Among stakeholders, the consensus was that a measure focused on the utilization of respiratory conditions was important and could be a useful tool for antibiotic stewardship efforts. During the public comment period, 80 comments were received from a range of stakeholders, including health plan representatives and clinicians, and nearly all commenters supported the proposed measure. Overall, commenters noted that respiratory conditions are a key target for antibiotic stewardship efforts. Feedback suggested that the measure will be useful for reporting and improving antibiotic prescribing in outpatient settings. The multistakeholder advisory panel concluded that the measure was relevant and useful, particularly if used alongside the existing condition-focused HEDIS measures.

### Final measure

The measure as finalized assesses the proportion of outpatient episodes for members 3 months of age and older with a diagnosis of a respiratory condition that resulted in an antibiotic prescription dispensed. The measure excludes episodes for which members had a competing diagnosis or comorbid condition for which antibiotics may appropriately be used.

## Discussion

The measure development process confirmed that antibiotic dispensing for respiratory conditions is a relevant and feasible indicator across a health plan’s membership and that there is variation that health plan administrators may wish to monitor as part of antibiotic stewardship efforts. Among the millions of respiratory condition encounters that occur annually, approximately one-third resulted in an antibiotic prescription, with variation across age- and product-line–based populations. Some degree of diagnosis variation across populations is expected given the epidemiology of respiratory conditions. However, variation may also reflect differential diagnosis practices driven by factors outside clinical relevance, such as institutional policies, ambiguous clinical guidelines, financial pressures, patient demand, and professional training.^22,23^ For example, variations in diagnosis coding have been documented between conditions that should or should not be treated with antibiotics.^24^ Although provider prescribing practices may vary, some studies suggest that coding bias may play a role in overprescribing, allowing providers to diagnose antibiotic-appropriate conditions at higher rates to avoid scrutiny when prescribing antibiotics.^24^ Inconsistency in diagnosis practices limits the ability to assess achievements in antibiotic stewardship for targeted clinical conditions.

The construction of the measure as a utilization metric was debated. During the conceptualization phase, there was discussion of the usefulness of a measure that assesses antibiotic “tiers,” which split the respiratory diagnoses into categories by which antibiotics are indicated. Tier 1 would include diagnoses for which antibiotics are almost always indicated; tier 2 would include diagnoses for which antibiotics may be indicated; and tier 3 would include diagnostic categories for which antibiotics are not indicated, or the indication was unclear.^10^ Such tiers have been used in studies measuring antibiotic appropriateness. In field testing, following this structure, an estimated 30% of outpatient, oral antibiotic prescriptions may have been inappropriate, which aligns with previous findings.^14^ However, during measure development, it was determined that a measure using a tiered system would be difficult to interpret because prescribing across the tiers can be less straightforward.

A utilization measure that simultaneously captures conditions for which antibiotics are inappropriate in addition to those for which antibiotics are appropriate also required a degree of clarification and communication. For example, during public comment, some commenters misunderstood the intent of the measure and sought further clarification. Commenters expected that higher utilization of antibiotic prescribing should be interpreted as “poor” performance. A measure of antibiotic utilization for all respiratory conditions does not provide an indication of “good” or “bad” performance for the outpatient network of a health plan. However, when used in combination with other HEDIS measures assessing antibiotic prescribing for bronchitis, pharyngitis, and upper-respiratory infection, a global barometer of antibiotic stewardship progress can be obtained. For example, whereas inappropriate antibiotic prescribing rates for bronchitis may be decreasing (suggesting positive achievements in stewardship), if overall antibiotic utilization for respiratory conditions more broadly is rising simultaneously, then further examination may be warranted. The advisory panel concluded that inclusion of both types of prescribing ensures that the measure is less vulnerable to variations in coding and diagnostic practices.

This study had several limitations. First, testing was conducted using 2018 and 2019 data. This period preceded the COVID-19 pandemic, which was declared in March 2020. After testing was complete, and based on stakeholder input, COVID-19 diagnosis codes were added to the measure. Analyses of 2021 performance data showed that while the rate of antibiotic utilization was lower compared to 2019, the age and sex distribution of the eligible population and the variation in measure performance across plans were similar to those shown in 2019. Related to COVID-19, it is possible that some stakeholders who would otherwise have commented on the draft measure during the public comment period may not have done so due to the overwhelming burden of the pandemic. However, the number of comments received was similar to numbers observed in previous public comment periods for past measure development projects.

The measure pertaining to antibiotic prescribing for respiratory conditions received support from health plan administrators and leaders in antibiotic stewardship as a tool for monitoring antibiotic utilization and as a flag for further analysis into the organization’s prescribing practices. Stakeholders indicated strong support for the new measure and encouraged improved antibiotic stewardship at the health-plan level, including the integration of the new measure into quality reporting programs. Given their financial role in the provision of health care and the data available to them, health plan administrators are well positioned to monitor antibiotic stewardship efforts across various outpatient settings. As such, the Centers for Disease Control and Prevention released a tool kit of strategies that health plan administrators can employ to improve outpatient antibiotic prescribing.^25^ As the COVID-19 pandemic continues to challenge stewardship efforts across conditions, improved quality metrics will be an important tool to ensure high-quality care for patients.
